# Regulatory Challenges and Frameworks for Fog Computing in Healthcare

**DOI:** 10.7759/cureus.66779

**Published:** 2024-08-13

**Authors:** Naveen Jeyaraman, Swaminathan Ramasubramanian, Sankalp Yadav, Sangeetha Balaji, Sathish Muthu, Madhan Jeyaraman

**Affiliations:** 1 Orthopaedics, ACS Medical College and Hospital, Dr MGR Educational And Research Institute, Chennai, IND; 2 Orthopaedics, Government Medical College, Omandurar Government Estate, Chennai, IND; 3 Medicine, Shri Madan Lal Khurana Chest Clinic, New Delhi, IND; 4 Orthopaedics and Traumatology, Orthopaedic Research Group, Coimbatore, IND; 5 Biotechnology, Karpagam Academy of Higher Education, Coimbatore, IND; 6 Orthopaedics, Government Medical College, Karur, IND; 7 Clinical Research, Virginia Tech India, Dr MGR Educational And Research Institute, Chennai, IND

**Keywords:** real-time analytics, regulatory challenges, data privacy, healthcare, fog computing

## Abstract

The integration of fog computing into healthcare promises significant advancements in real-time data analytics and patient care by decentralizing data processing closer to the source. This shift, however, introduces complex regulatory, privacy, and security challenges that are not adequately addressed by existing frameworks designed for centralized systems. The distributed nature of fog computing complicates the uniform application of security measures and compliance with diverse international regulations, raising concerns about data privacy, security vulnerabilities, and legal accountability. This review explores these challenges in depth, discussing the implications of fog computing's decentralized architecture for data privacy, the difficulties in achieving consistent security across dispersed nodes, and the complexities of ensuring compliance in multi-jurisdictional environments. It also examines specific regulatory frameworks, including Health Insurance Portability and Accountability (HIPAA) in the United States, General Data Protection Regulation (GDPR) in the European Union, and emerging laws in Asia and Brazil, highlighting the gaps and the need for regulatory evolution to better accommodate the nuances of fog computing. The review advocates for a proactive regulatory approach, emphasizing the development of specific guidelines, international collaboration, and public-private partnerships to enhance compliance and support innovation. By embedding privacy and security by design and leveraging advanced technologies, healthcare providers can navigate the regulatory landscape effectively, ensuring that fog computing realizes its full potential as a transformative healthcare technology without compromising patient trust or data integrity.

## Introduction and background

The integration of fog computing into healthcare systems represents a transformative departure from centralized data processing architectures towards more distributed, edge-centric models [1]. 

Fog computing is an extension of cloud computing that brings computation, storage, and networking capabilities closer to the data source, typically at the network edge [2]. It acts as an intermediary layer between end devices and traditional cloud data centers, enabling faster processing and reduced latency. In healthcare, fog computing facilitates real-time data analysis, enhances responsiveness in critical situations, and supports the growing ecosystem of Internet of Medical Things (IoMT) devices [1,3,4]. Fog computing offers several significant advantages over traditional cloud-based methods in healthcare which are as follows: (i) Reduced latency: By processing data closer to the source, fog computing dramatically reduces response times, which is crucial for real-time health monitoring and emergency response systems; (ii) Bandwidth conservation: Fog nodes can filter and process data locally, sending only relevant information to the cloud, thus reducing network congestion and bandwidth usage; (iii) Enhanced privacy and security: Sensitive patient data can be processed locally, minimizing the exposure of raw data during transmission and storage in centralized cloud systems; (iv) Improved reliability: Fog computing can continue to function even with intermittent internet connectivity, ensuring continuous operation of critical healthcare systems; (v) Scalability: It allows for easier integration of numerous IoMT devices, supporting the growing trend of personalized and home-based healthcare; (vi) Cost-effectiveness: By reducing the need for constant cloud communication and storage, fog computing can lower operational costs for healthcare providers; (vii) Context-awareness: Fog nodes can adapt to local conditions and patient needs, enabling more personalized and efficient healthcare delivery. These advantages make fog computing particularly valuable in scenarios requiring real-time analytics, rapid response, and handling of large data volumes from distributed sources, such as remote patient monitoring, emergency services, and large-scale health data analysis [5,6].

**Figure 1:**
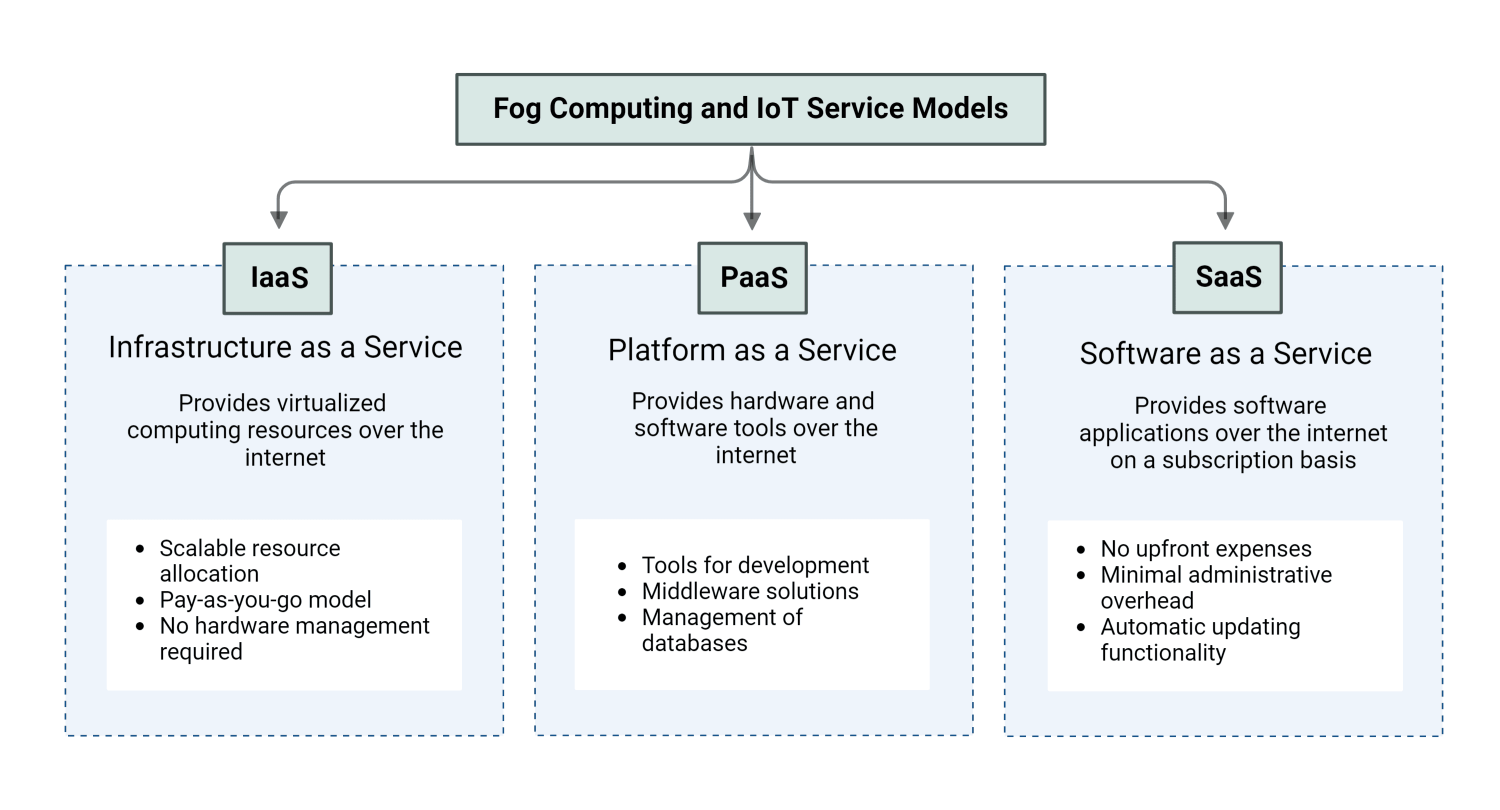
Service models of fog computing for healthcare IoT: Internet of things Image Credit: Dr. Madhan Jeyaraman

This technological advancement holds the potential to augment real-time data analytics and patient care by bringing computational capabilities closer to data origins, thereby diminishing latency and enhancing response times in critical healthcare applications. Despite the evident benefits of fog computing, its deployment within the sensitive realm of healthcare raises notable concerns pertaining to regulation, privacy, and security, demanding meticulous scrutiny. Presently, healthcare data management systems predominantly rely on centralized cloud computing frameworks, which adhere to established regulatory compliance measures such as the Health Insurance Portability and Accountability Act (HIPAA) in the United States [7] and the General Data Protection Regulation (GDPR) in the European Union [8]. However, these regulations are primarily tailored for centralized data processing infrastructures and do not comprehensively address the intricacies introduced by the decentralized, multi-node architecture of fog computing. Consequently, there exists a significant knowledge disparity in regulatory frameworks capable of effectively governing the distinctive attributes of fog computing, including multi-jurisdictional data flow, node-level data processing, and real-time cross-border data exchanges. The currently available models of fog computing services are given in Figure 1.

The existing privacy and security protocols for traditional cloud computing do not seamlessly translate to fog computing architectures. The dispersed nature of fog nodes introduces new challenges in ensuring uniform security measures across all nodes and complying with diverse international laws and standards, especially when these nodes are located in different legal jurisdictions [9]. This decentralized approach complicates data privacy, with issues such as consent management and data integrity becoming more difficult to manage and enforce. The rapid evolution of technology outpaces the current regulatory frameworks, creating a gap between technological capabilities and legal safeguards, thereby increasing the risk of privacy breaches and data misuse [10]. Given these challenges, we aim to explore the regulatory, privacy, and security challenges of integrating fog computing into healthcare.

## Review

Overview of regulatory challenges in incorporating fog computing within healthcare

**Figure 2:**
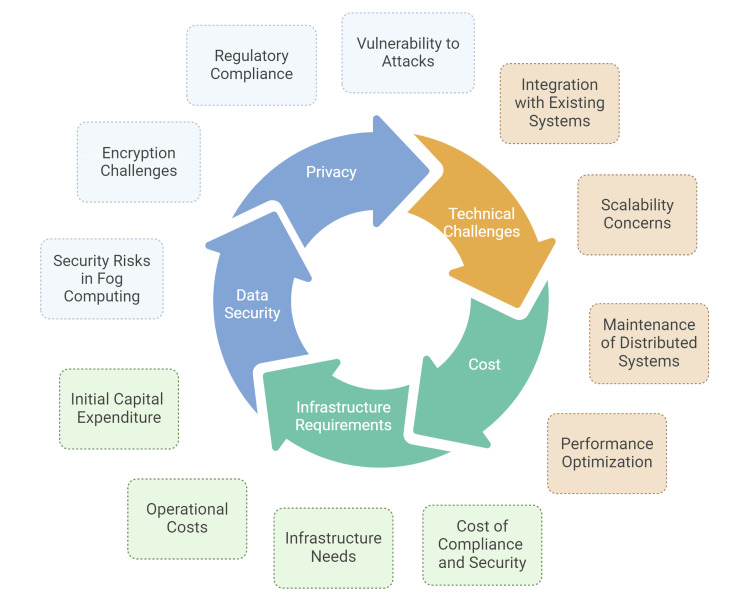
Challenges with fog computing in healthcare Image Credit: Dr. Sankalp Yadav

Fog computing in healthcare represents a transformative shift from centralized to decentralized data processing, offering real-time analytics and enhanced patient care [11-13]. However, this shift also introduces complex regulatory challenges, particularly in the realms of data privacy, security, and legal compliance. The intricacies of these challenges require a nuanced understanding and a proactive approach to regulatory framework development as shown in Figure 2.

Data Privacy

In healthcare, where patient data is particularly sensitive, the shift toward fog computing raises substantial data privacy concerns. The primary challenge lies in the decentralized nature of fog computing, where data is processed on various nodes that may not all adhere to the same privacy standards [14,15]. This decentralization makes it inherently difficult to apply uniform privacy protections across all nodes involved in the processing of healthcare data. For instance, different nodes in the fog computing architecture could be located in different jurisdictions, each with its own privacy laws and standards. This geographical and jurisdictional diversity can lead to inconsistencies in data handling and risk breaches of privacy.

The situation becomes even more complex when considering international data transfers in fog environments, where data might move between nodes across borders in real time. Fog computing complicates the enforcement of consent management. In traditional centralized systems, it is easier to manage patient consent for data usage since the data flows through a single point. In contrast, in fog computing, data might be processed and stored temporarily across multiple nodes, making it challenging to ensure that all processing adheres to the patient's consent parameters. This issue necessitates developing more dynamic and granular consent mechanisms that are capable of functioning effectively in a decentralized setup [2,6]. Incorporating a decentralized consent management framework is essential. This framework should include mechanisms for obtaining, verifying, and managing patient consent at every node. Implementing blockchain technology can ensure an immutable record of consent across all nodes, enhancing transparency and traceability. Dynamic consent mechanisms can leverage smart contracts within blockchain frameworks to automatically enforce consent parameters as data moves through different nodes. Additionally, integrating identity management solutions that provide federated authentication can help in verifying patient consent across multiple jurisdictions.

Security Challenges

Security in fog computing for healthcare is pivotal yet problematic to enforce consistently across a dispersed network of nodes. Each node represents a potential entry point for security threats, which could compromise the entire network. The security measures implemented in traditional cloud computing are not directly transferable to fog computing due to the different architectural and operational dynamics. One significant security challenge is ensuring the integrity and confidentiality of health data as it moves between nodes in the fog [16]. Since data processing occurs at the edge, closer to data sources, there is a greater risk of interception or alteration [16,17]. Implementing robust encryption protocols and secure data transmission channels is critical, but these must be optimized for low latency to meet the real-time processing needs of healthcare applications. Additionally, the physical security of fog nodes is a concern [18]. Unlike data centers used in cloud computing, fog nodes are often located in accessible areas that may be vulnerable to tampering or theft. Ensuring the physical security of these nodes is crucial to preventing unauthorized access to stored data and the potential for tampering with healthcare data processing.

A critical challenge in fog computing for healthcare is the question of data validation across multiple systems and nodes [19]. As data passes through various points in the fog architecture, it raises the important issue of how many times and at which stages the data needs to be validated to ensure its integrity and accuracy. This challenge is particularly significant in healthcare, where data integrity directly impacts patient care and safety. Multiple validation points may be necessary to detect any corruption or unauthorized alterations as data moves through the system. However, excessive validation could potentially introduce latency, negating some of the speed benefits of fog computing. Striking the right balance between thorough validation and maintaining efficient data flow is crucial. Healthcare providers and fog computing solution developers must collaboratively establish clear protocols for data validation, potentially incorporating blockchain or other distributed ledger technologies to maintain an immutable record of data integrity throughout its journey across the fog network [20]. These protocols should define validation frequency, methods, and responsible parties at each stage of data transmission and processing, ensuring that data remains reliable and trustworthy from point of origin to final use.

Compliance with Laws and Regulations

Compliance with existing healthcare regulations, such as HIPAA in the United States, GDPR in Europe, or other national laws, is a non-trivial task in fog computing environments [7,8]. These laws were generally designed with more centralized data processing architectures in mind. As such, applying these laws to the decentralized, multi-node environment of fog computing often results in ambiguity regarding compliance responsibilities. Healthcare providers and fog service operators must navigate these legal complexities while ensuring compliance at every node within their network [21]. This challenge is compounded by the continuous evolution of both technology and regulatory landscapes, requiring ongoing adjustments to compliance strategies. For example, GDPR imposes strict requirements on data controllers and processors, including the need for detailed documentation of data processing activities and maintaining high standards of data protection by design and by default.

The variability in compliance across different jurisdictions also complicates the deployment of uniform fog computing solutions in healthcare. A healthcare provider operating across multiple countries must ensure that their fog computing infrastructure complies with the varying requirements of each jurisdiction, which can differ significantly in terms of data protection, data sovereignty, and patient rights [22]. In addressing these regulatory challenges, stakeholders in healthcare fog computing must advocate for and contribute to the development of clear, specific regulatory frameworks that consider the unique aspects of fog computing. These frameworks should aim to protect patient privacy, ensure data security, and facilitate compliance with existing laws while also being flexible enough to adapt to future technological advancements in fog computing and healthcare. Engaging in this proactive regulatory dialogue is essential for leveraging the full potential of fog computing to enhance patient care without compromising on legal or ethical standards.

Specific regulatory frameworks

United States: HIPAA and Health Information Technology for Economic and Clinical Health (HITECH)

In the United States, healthcare data regulation primarily revolves around HIPAA, which establishes nationwide standards for safeguarding health information [7]. HIPAA applies to entities and associates involved in handling protected health information (PHI), mandating the implementation of physical, administrative, and technical measures to protect data. Fog computing systems must align with HIPAA's security and privacy provisions, particularly in ensuring the secure transmission of PHI between fog nodes and other entities.

The HITECH Act expands upon HIPAA's mandates, imposing stricter penalties for non-compliance and extending obligations to healthcare provider associates [23]. HITECH promotes the adoption of electronic health records (EHR) and facilitates secure health information exchange through enhanced data protection mechanisms, crucial in fog computing contexts. The United States Food and Drug Administration (FDA) plays a pivotal role in regulating medical devices incorporating fog computing technologies, especially those employed in real-time health monitoring and diagnostics [24,25]. The FDA has issued directives categorizing software as a medical device (SaMD), encompassing software functionalities within fog computing architectures. These directives necessitate manufacturers to ensure their devices meet safety and efficacy standards, requiring thorough testing and validation of fog computing components integrated into medical devices.

An important regulatory consideration is whether each fog computing component at the local level requires validation by the FDA. Currently, the FDA's approach to SaMD suggests that not every fog component would necessarily require individual validation [26]. Instead, the FDA is likely to focus on the overall system's functionality and its impact on patient safety and clinical decision-making. However, components that directly influence diagnostic or therapeutic decisions may require specific validation. Healthcare providers and technology developers should work closely with the FDA to determine which elements of their fog computing infrastructure fall under regulatory purview. This approach allows for innovation while ensuring critical components meet necessary safety and efficacy standards. As fog computing in healthcare evolves, the FDA may develop more specific guidelines to address the unique challenges posed by distributed computing architectures.

European Union: GDPR

The GDPR stands as a fundamental pillar of data protection within the European Union, significantly influencing the treatment of healthcare data [8]. GDPR bolsters individual privacy rights and imposes heightened responsibilities on organizations processing personal data. In the context of fog computing, GDPR mandates that data privacy be ingrained into system design from its inception, ensuring robust data protection measures. GDPR's requirement for explicit consent can be managed by implementing decentralized consent frameworks. For example, smart contracts within a blockchain can dynamically enforce and record patient consent, ensuring compliance even as data is processed and transferred across multiple nodes. This technology ensures that consent is obtained, verified, and respected in real time, maintaining GDPR compliance.

A pivotal challenge under GDPR pertains to the requirement for transparent data consent, necessitating that consent be freely given, specific, informed, and unequivocal. This poses complexities in fog environments where data processing occurs across multiple nodes, potentially complicating consent management procedures. Moreover, GDPR imposes limitations on data transfers outside the EU, necessitating that fog nodes situated beyond EU borders adhere to equivalent data protection standards [8]. Mechanisms such as Binding Corporate Rules (BCRs) or Standard Contractual Clauses (SCCs) may be employed to achieve compliance. The forthcoming ePrivacy Regulation, poised to supersede the existing ePrivacy Directive, supplements GDPR by concentrating on the confidentiality of electronic communications [27]. It incorporates provisions relevant to fog computing, including stringent consent requirements for storing and accessing information on end-user devices, which are often integral to fog computing networks [27]. Although not yet enacted, this regulation will further delineate the handling of healthcare data transmitted through fog computing networks.

Asia

In Asia, the regulatory approach to data protection and healthcare varies significantly across the region.

Japan: The recent amendments to Japan's Act on the Protection of Personal Information (APPI) signify a significant step towards aligning the nation's data protection regulations with international standards, notably the GDPR [28]. These modifications have bolstered individual rights and introduced stringent mandates concerning cross-border data transfers. Specifically relevant to healthcare entities leveraging fog computing networks, APPI now mandates organizations to implement the requisite security measures for safeguarding personal data. This directive directly impacts healthcare providers utilizing fog computing nodes, either within Japan or in possession of data from Japanese individuals. Compliance necessitates the meticulous integration of privacy controls within fog computing infrastructures to align with APPI's provisions [28].

China: China's Cybersecurity Law, alongside the more recent Personal Information Protection Law (PIPL), underscores the nation's commitment to safeguarding personal information and crucial data [29]. These regulations mandate critical data infrastructure operators to store domestically collected and produced personal data within China, a directive with direct ramifications for fog computing services handling healthcare data. Specifically, the Cybersecurity Law emphasizes the protection of critical information infrastructure, compelling operators to localize personal data collected within China [29]. This localization mandate can pose challenges for the deployment of cross-border fog computing services. Meanwhile, the PIPL, akin to the GDPR, elevates personal data protection standards and delineates consent requisites, thereby influencing how healthcare providers navigate patient data within fog computing frameworks.

India: India is in the process of implementing its Personal Data Protection Bill [30], which draws inspiration from GDPR. It addresses similar issues of consent, data localization, and the rights of individuals regarding their data. Compliance with this emerging framework will be essential for fog computing services operating in the healthcare sector within India.

Brazil

Brazil's Lei Geral de Proteção de Dados (LGPD) closely mirrors the GDPR and marks a substantial change in the data protection regulatory landscape within the nation [31]. Applicable to any entity processing individuals' data in Brazil, irrespective of location, the LGPD underscores the importance of consent for data processing and affords individuals significant rights concerning their data. In the realm of fog computing in healthcare, this necessitates that systems be structured to enable patients to effectively exercise their rights, including accessing, rectifying, and erasing their data. This mandate influences the handling of data across the dispersed nodes of fog computing networks. The comparison of data privacy regulations relevant to fog computing in healthcare is tabulated in Table 1.

**Table 1:** Comparison of data privacy regulations relevant to fog computing in healthcare HIPAA: Health Insurance Portability and Accountability Act; GDPR: General Data Protection Regulation; APPI: Act on the Protection of Personal Information; PIPL: Personal Information Protection Law; LGPD:  Lei Geral de Proteção de Dados; PHI: protected health information

Region/Country	Regulation	Key provisions impacting fog computing	Challenges in fog computing context
United States	HIPAA	Requires secure handling of PHI; mandates physical, administrative, and technical safeguards.	Difficulties in ensuring PHI security across dispersed fog nodes.
European Union	GDPR	Data protection by design and by default; strict transfer regulations.	Managing consent and data integrity across multiple nodes in different jurisdictions.
Japan	APPI	Enhanced protection for personal data; strict cross-border data transfer rules.	Ensuring all nodes comply with stringent data protection standards.
China	Cybersecurity Law and PIPL	Data localization; strong protection for personal and critical data.	Challenges with data localization and compliance in multi-node environments.
Brazil	LGPD	Strong emphasis on consent and data subject rights.	Integrating dynamic consent mechanisms across decentralized computing platforms.
India	(Proposed) Personal Data Protection Bill	Inspired by GDPR; includes data localization and consent requirements.	Harmonizing fog computing operations with evolving national standards.

Balancing innovation and compliance: fog computing in healthcare

**Figure 3:**
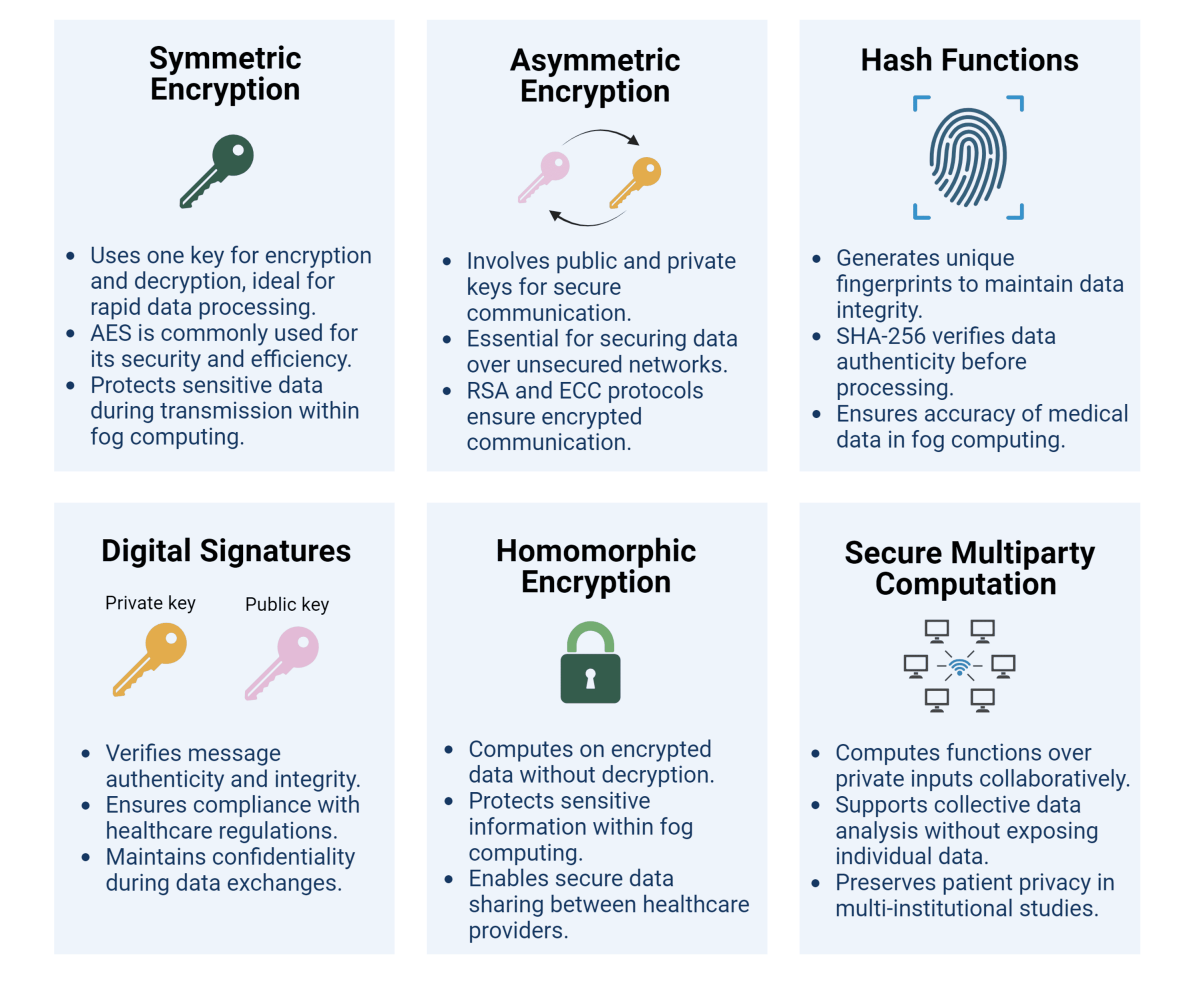
Security frameworks and protocols, cryptographic methods, used for data protection in fog computing AES: Advanced Encryption Standard; RSA: Rivest, Shamir, Adleman; ECC: Elliptic Curve Cryptography; SHA-256: Secure Hash Algorithm: 256 Image Credit: Dr. Naveen Jeyaraman

Fog computing stands as a notable advancement in healthcare technology, promising improved patient care by facilitating real-time data processing and analytics at the network edge. A study has shown that fog computing can reduce data processing latency by up to 90% compared to traditional cloud computing models. This significant decrease in latency allows for more rapid processing of health data, which is crucial in emergency medical scenarios and real-time patient monitoring systems. For instance, processing health data at the edge rather than transmitting it to a centralized cloud reduces the round-trip time and ensures timely responses to critical health events [32].​ Nonetheless, the implementation of these technologies faces the intricate task of navigating rigorous regulatory standards aimed at safeguarding patient privacy and upholding data security. Striking a balance between fostering innovation and adhering to compliance measures poses a pivotal challenge for healthcare providers. The current security frameworks and protocols in place for data protection using fog computing are given in Table 2 and Figure 3.

**Table 2:** Security measures for fog computing in healthcare

Security aspect	Technology/Method	Purpose and application in fog computing
Data Encryption	Advanced Encryption Standard (AES)	To secure data transmission between fog nodes and prevent unauthorized access.
Integrity Checks	Blockchain Technology	To maintain and verify the integrity of data across the decentralized network.
Access Control	Role-Based Access Control (RBAC)	To limit access to data based on user roles, crucial in dispersed node environments.
Real-Time Monitoring	AI-based anomaly detection	To identify and respond to security threats in real-time across the network.
Physical Security	Tamper-resistant hardware and secure enclosures	To protect fog nodes located in accessible areas from physical tampering.
Compliance Auditing	Automated compliance tools	To continuously monitor and ensure adherence to relevant legal and regulatory standards.

Emphasizing Privacy by Design

One of the most effective strategies for balancing innovation with compliance is the adoption of the "privacy by design" approach. This concept, which has been formalized within the GDPR in Europe, requires that data protection safeguards be embedded directly into the design of technologies at an early stage rather than being added on as an afterthought. In the context of fog computing, this means integrating strong encryption measures, secure data transmission protocols, and access controls right from the initial phases of technology development. For healthcare providers, implementing privacy by design involves conducting thorough risk assessments to identify potential privacy risks associated with deploying fog computing technologies. This should also include the development of decentralized consent management systems. These systems can utilize blockchain technology to create an immutable ledger of consent records, ensuring that patient consent is obtained and honored across all nodes. By integrating these systems from the outset, healthcare providers can ensure compliance with regulations such as HIPAA and GDPR. This process also includes developing clear policies and procedures that address how data is collected, processed, stored, and shared across the fog computing network. By embedding these privacy features directly into the fog computing architecture, healthcare providers can ensure compliance with laws like HIPAA in the United States [7] and GDPR in the European Union [8], thereby protecting patient data while harnessing the benefits of real-time data analytics.

Leveraging Advanced Technologies for Security

Security is a paramount concern when deploying fog computing solutions in healthcare due to the sensitive nature of health data. Advanced technological solutions such as blockchain and artificial intelligence (AI) can play a pivotal role in enhancing the security of fog computing architectures [20,33]. Blockchain technology, for example, can be used to create decentralized and immutable ledgers of all transactions and data exchanges within the fog computing network. This capability provides an additional layer of security and transparency, making it easier to track and verify the integrity of data being processed at the edge of the network. Moreover, AI can be utilized to monitor and manage data flows within the fog computing system, detecting anomalies that may indicate a security breach or unauthorized data access. By integrating AI-driven security tools, healthcare providers can proactively manage risks and respond to threats in real time, thus maintaining the integrity and confidentiality of patient data across the network.

Developing a Compliance Culture

To effectively reconcile innovation with compliance, healthcare entities must foster a culture of adherence to regulatory standards that permeates throughout the organization. This entails providing comprehensive training and education to all staff members regarding the significance of data protection and the specific compliance obligations associated with fog computing technologies. A well-informed workforce is less prone to instigate data breaches and more inclined to uphold optimal practices for data management and security. Healthcare providers can establish dedicated compliance units charged with overseeing the integration of fog computing technologies, ensuring alignment with regulatory mandates [34]. These teams should collaborate closely with information technology (IT) departments, legal counsel, and external specialists to remain abreast of evolving regulatory landscapes and technological advancements.

Regular Audits and Continuous Improvement

Regular auditing is critical to ensure ongoing compliance with regulatory requirements in a fog computing environment. Audits help identify any shortcomings in the current data protection strategies and provide insights into how these strategies can be improved. Healthcare providers should conduct regular internal and external audits of their fog computing systems, focusing on both the technical and procedural aspects of data protection. Continuous improvement is also essential. As both technology and regulatory landscapes evolve, healthcare providers must be willing to adapt and update their fog computing systems to address new challenges and opportunities. This might involve upgrading technology, refining operational processes, or revising compliance protocols to meet new or amended regulations [35,36].

Recommendations for policymakers and providers

For Policymakers: Enhancing Regulatory Frameworks

Develop specific guidelines for Fog Computing: Policymakers should create clear, specific guidelines that address the unique aspects of fog computing. These guidelines should cover data privacy, security measures, and compliance checks specific to the decentralized nature of fog computing. This will help in removing ambiguities that currently exist due to the application of general data protection laws to fog computing scenarios.

Promote international collaboration: Given the global nature of data flows and the technological deployment of fog computing, international collaboration is crucial. Policymakers should work towards harmonizing regulations across borders to facilitate seamless, secure data exchange that complies with international standards. This collaboration could be in the form of treaties, agreements, or joint frameworks that recognize and reconcile the differences in national laws.

Encourage public-private partnerships: To foster innovation while ensuring security and compliance, policymakers should promote partnerships between government bodies, technology providers, and healthcare organizations. These partnerships can lead to the development of more robust and compliant fog computing solutions by leveraging private-sector innovation and public regulatory oversight.

Invest in regulatory technology (RegTech): Policymakers should invest in developing and supporting RegTech solutions that can help manage the complexity of compliance in fog computing environments. These technologies can automate compliance processes, ensure continuous monitoring, and provide real-time analytics to assist both providers and regulators in maintaining compliance.

For Providers: Ensuring Compliance While Innovating

Implement robust risk management processes: Providers should establish comprehensive risk management protocols that address the specific risks associated with fog computing. This includes conducting regular risk assessments to identify potential vulnerabilities in the network and implementing appropriate mitigation strategies. Risk management should be an ongoing process, with strategies updated regularly to respond to new threats and changes in the regulatory landscape.

Develop decentralized consent management systems: Policymakers should endorse the development and adoption of decentralized consent management systems using blockchain and smart contracts. These systems can automate consent verification and enforcement at each node, ensuring that patient consent is respected throughout the data processing lifecycle.

Adopt interoperable and scalable solutions: When implementing fog computing architectures, healthcare providers should opt for solutions that are not only compliant with current regulations but are also scalable and interoperable. This ensures that the technology can adapt to future changes in regulations and can seamlessly interact with other systems and technologies, thereby reducing the risk of non-compliance due to system incompatibilities.

Focus on data governance and ethical use: Providers must develop strict data governance policies that define how data is collected, used, stored, and deleted in the fog computing environment. These policies should align with ethical guidelines and compliance requirements, ensuring that patient data is handled responsibly. Clear data governance helps in maintaining transparency and trust, both crucial for patient relations and regulatory compliance.

Train and educate staff regularly: Ongoing training and education programs for all staff members involved in the operation of fog computing systems are vital. These programs should focus on the importance of compliance, the specifics of fog computing operations, and updates on new regulations or technological advances. Well-informed staff are less likely to commit breaches and more likely to contribute to the secure and compliant use of technology.

Engage in continuous monitoring and auditing: Sustained monitoring of the fog computing infrastructure is indispensable for promptly identifying and addressing potential compliance lapses or security vulnerabilities. Healthcare providers should conduct routine audits of their systems to verify compliance and pinpoint opportunities for enhancement. This proactive stance not only supports ongoing compliance but also bolsters the overall security and efficacy of fog computing operations. The stakeholder responsibilities in implementing fog computing in healthcare are depicted in Table 3.

**Table 3:** Stakeholder responsibilities in implementing fog computing in healthcare HIPAA: Health Insurance Portability and Accountability Act; GDPR: General Data Protection Regulation

Stakeholder	Responsibilities	Impact on Fog Computing Implementation
Healthcare Providers	Ensure compliance with HIPAA, GDPR, etc.; implement privacy by design; regular staff training.	Direct control over patient data and primary responsibility for compliance.
Technology Providers	Develop secure and compliant fog computing solutions; provide ongoing support and updates.	Essential in designing and maintaining the infrastructure that adheres to regulations.
Regulators	Formulate specific guidelines for fog computing; enforce compliance.	Set the legal framework and oversee adherence to it, influencing how technologies are deployed.
Patients	Exercise data rights; provide informed consent.	Their participation and awareness are crucial for ethical data-handling practices.
International Collaborators	Engage in treaties and agreements to harmonize regulations.	Facilitate cross-border data flows and cooperative regulatory environments.

Developing Specific Regulatory Frameworks

The necessity of developing specific regulatory frameworks for fog computing in healthcare is both evident and essential. In the United States, bodies like the FDA, alongside the European Medicines Agency (EMA) in the European Union and similar organizations globally, should spearhead this initiative. These agencies possess the requisite authority and expertise to enact binding regulations that address the unique challenges and opportunities presented by fog computing. The inclusion of healthcare technology experts is crucial. Their deep understanding of fog computing applications in healthcare will ensure that the regulations are not only technologically sound but also feasible. Similarly, healthcare providers such as hospitals and clinics can offer valuable insights into practical implementation challenges and their implications on patient care. Legal experts specializing in healthcare law and data protection are also vital to this process. Their involvement ensures that new regulations are in harmony with existing laws and adequately address emerging legal issues. Furthermore, incorporating the views of patient advocacy groups guarantees that patient rights and privacy are vigorously protected in these new frameworks. Industry representatives from companies that develop fog computing solutions should also have a seat at the table. Their perspective is indispensable for understanding innovation trajectories and practical implementation considerations. Additionally, international organizations like the World Health Organization (WHO) and the International Medical Device Regulators Forum (IMDRF) can contribute to creating a harmonized regulatory approach across different jurisdictions.

The process of developing these regulations should commence with comprehensive research into existing frameworks and identifying the gaps specifically related to fog computing. Public consultations will allow for the incorporation of diverse perspectives, making the regulations more comprehensive and robust. Pilot programs are crucial for testing these regulations in real-world settings, ensuring their effectiveness before full-scale implementation. Finally, regular reviews and updates must be integral to the regulatory process, adapting to ongoing technological advancements to maintain relevance and efficacy. The ultimate goal is to forge a framework that effectively addresses data privacy, security protocols, interoperability standards, accountability measures, and cross-border data flow, ensuring that fog computing's potential in healthcare is maximized while safeguarding patient data and safety.

## Conclusions

The integration of fog computing into healthcare systems presents a complex interplay of innovation and compliance challenges. As healthcare providers seek to leverage the benefits of real-time data processing at the network edge, they must also navigate a labyrinth of regulatory requirements designed to protect patient privacy and data security. The development of specific regulatory frameworks that address the unique aspects of fog computing is essential for mitigating risks associated with data privacy, security breaches, and cross-jurisdictional compliance. Furthermore, fostering a culture of compliance, emphasizing privacy by design, and employing advanced security technologies are pivotal strategies for ensuring that the potential of fog computing is realized without compromising ethical or legal standards. As technology evolves, continuous dialogue among policymakers, healthcare providers, and technology experts is crucial to refine and adapt regulatory approaches, ensuring that fog computing can be both a transformative healthcare tool and a model of regulatory compliance.
